# Korea’s low birth rate issue and policy directions

**DOI:** 10.4069/kjwhn.2021.02.16

**Published:** 2021-03-24

**Authors:** Kyung Ae Cho

**Affiliations:** Secretary (2018-2021), Korea Population, Health and Welfare Association, Seoul, Korea

## Introduction

In early 2021, the South Korean media poured out discouraging articles on the natural decrease in the nation’s population. The low birth phenomenon has continued for almost two decades since 2002, when the total fertility rate (TFR)—defined as the average number of children that a woman would bear during her reproductive lifespan between ages 19 and 49 years—dropped below 1.3 [1]. In 2020, the number of births was surpassed by deaths, causing a natural population decline. To make the matter worse, young people are more likely to delay marriage or having children in the coronavirus disease 2019 (COVID-19) era, leading to an even lower number of expected births this year. It is a national task to slow the trend of low birth rates and an aging population by taking appropriate actions to bring about social, economic, cultural, and regional changes that can create a more sustainable society. In this paper, I would like to discuss the current status of low birth in South Korea (hereinafter, Korea) as well as the issues and future directions of the country’s population policy.

## How serious is the birth issue in Korea?

The number of births in Korea has declined dramatically on three occasions since the end of the Korean War. The country boasted high fertility rates from the end of the Korean War until 1982, where the number of annual births was maintained between 800,000 and 1 million. In 1983, the nation’s TFR dropped below the population replacement level of 2.1, thanks to the government’s strong fertility regulation policy, widespread use of contraception, and normalization of smaller family sizes [1]. This is referred to as the first population change in Korea. Between 1983 and 2000, the number of annual births stayed at around 600,000; as such, this period has been labeled the low birth stage. In 2001, the number of births dropped dramatically to 400,000, with a TFR of 1.3 [1], corresponding to the second population change. The 1997 Asian financial crisis seems to have had ripple effects on the trend of individuals delaying marriage and subsequently having children. Low fertility continued through 2016, when the number of births further plunged to 300,000 (the third population change). In 2019, Korea became the only country in the world where a woman is expected to give birth to less than one child in her life, with a TFR of 0.92 [1].

The government has not been complacent regarding the issue of low birth. In 2005, the government enacted the Framework Act on Low Birth Rate in an Aging Society and organized the Presidential Committee on Ageing Society and Population Policy [2] which is led by the president and consists of ministers and experts, as very low birth rates have continued since 2001 with a TFR under 1.3. The government has renewed the Basic Plan for Aging Society and Population Policy every 5 years since 2006 and implemented policies across all walks of society. The first and second rounds of the plan included policies and measures to support pregnancy and childbirth, as well as childcare services to relieve couples’ financial burden of raising children. Starting in the third round, which came into effect in 2015, additional support was provided for job-seeking and housing for newlyweds to address the financial factors that contribute to getting married later and staying single longer. The government revised the third round in 2018, announcing the Policy Roadmap for an Inclusive Nation [3]. The new plan designed a transition to a policy aimed at improving the quality of life for all generations based on the criticism that previous plans have simply focused on the government’s role in encouraging childbirth without putting people at the center. Despite such efforts, the fertility rate seems far from ready to bounce back.

## Why aren’t young Koreans having children?

When asked if they agreed with the statement “being a parent is an invaluable thing in life,” most Koreans (81.7%) agreed, including 67.0% of people in their 20s [4]. All age groups, including those in their 20s, reported thinking that having two children is ideal. In reality, the younger generation in Korea make dark jokes about having to give up three things in life, i.e., so-called "*sam po*"—dating, marriage, and having children. They feel like they do cannot afford to spare any time, energy, or emotions that should go to surviving fierce competition in school and building qualifications to land a job in the tight job market. Korea has become a society where young people find it difficult to plan for a life where securing a job leads to getting married and having a family, because they are too anxious about obtaining a secure job, a stable income, and a place to live.

Exploring differences between men and women in how they see marriage and family may also provide insights into this phenomenon. The traditional gender roles of male breadwinners and female housekeepers are being challenged. A survey by the Korean Population, Health and Welfare Association [5] showed that six out of 10 single 30-somethings said they would like to get married. Two out of 10 responded they did not want to get married and the other two said they were not sure. Regarding the reason why they were hesitant to get married, 51.1% of male respondents said they found it difficult to agree on marriage terms such as housing and finances. Meanwhile, 25.3% of female respondents said they were “happy living alone,” and 24.7% said they were hesitant to tie the knot because of “the culture of patriarchy and gender inequality.” Five out of 10 respondents were positive about having children, while negative opinions were expressed through responses of “not [being] confident if I can do well in parenting” (24.6%), and “the financial burden of childcare” (24.3%).

In a 2019 survey by the Korean Women’s Development Institute on the priority of developmental tasks in life among young women and men in their 20s-30s [6], female respondents placed a higher priority on work and personal life than partnership and children. The results were not markedly different from those of their male peers. This shows that today’s young women choose to prioritize their career throughout their life cycle, as do young men.

A high percentage of young women also agreed that their partners’ participation in childcare, equal distribution of household chores, and partner’s maternity/paternity leave are prerequisites for them to consider having children. This is sharply distinct from the responses of their male counterparts, who pointed to their own financial situation and stable job as the biggest factors. This means that today’s young women will not tolerate traditional gender roles or unequal treatment, and would only choose to have children if their partners actively share the burden, allowing them to keep their careers without facing an existential crisis. These results provide a valuable perspective on what kind of environment is needed for today’s young women.

## How should we approach low birth policies?

The international community has been discussing the most suitable way to approach population policies for a long time. In the 1970s and 1980s, governments around the world implemented strong population policies focused on family planning. Discussions were held about whether the population should be regulated in light of how population growth seemed to deter economic development. At the 1994 Cairo International Conference on Population and Development and the 1995 Beijing World Conference on Women, the paradigm for population policies shifted from controlling population size to acknowledging human rights [7].

The 1994 Cairo Conference confirmed that population policies should value the unique life of human beings rather than trying to regulate population size, acknowledging that reproductive rights are basic human rights. Governments committed to goals to be met by 2015 to ensure universal access to reproductive health services, such as reducing infant/child/maternity deaths, universal education, and family planning to help empower women and improve their social status. The 1995 Beijing Conference advised governments to protect women’s rights to reproductive health without unwanted pregnancy and birth, recognizing that women play a key role in socioeconomic development. Reproductive health and rights refer to the state of physical, mental, psychological, and social well-being free from illness, malfunctions, and/or disabilities. This acknowledgement helped the paradigm of population policy to move away from emphasizing economic development issues and to prioritize individual rights, health, and welfare. Since then, the global community has started to understand sexual and reproductive health and rights as part of human rights, and has protected comprehensive services that provide information, counseling, education, and healthcare.

How did this issue play out in Korea? The Korean government continued to implement measures to control population growth for a considerable time even after reaching its goal of curbing population growth by 1983, earlier than it had planned. It was only in 1996 that the fertility regulation policy was replaced by the goal of “improving the quality of the population.” Concrete actions were taken to promote reproductive health such as providing sex education for teenagers and supporting mothers, infants, and children. With plummeting birth rates in the beginning of the 2000s, the government started to implement policies to encourage childbirth. Based on the diagnosis that the nation’s low birth and population aging trends will aggravate social and economic crises, authorities put measures in place to promote childbirth and support childcare to achieve increased fertility [8]. However, not enough efforts were made to enhance gender equality, understand childbirth as women’s individual right, and provide support for health and welfare. The younger generation saw this kind of policy as the government attempting to enforce traditional cultural norms by demanding that they have children. Korean women have expressed their repulsion against recent initiatives like the disclosure of the “birth map” and conducting the National Survey on Fertility and Family Health and Welfare, claiming that the government simply sees women as a tool for childbirth and is attempting to control population growth [9].

## A paradigm shift is needed in low birth policy

The Korean government released the Fourth Basic Plan for Ageing Society and Population Policy for the 5-year period beginning in 2021 [2]. The plan demonstrates a shift in paradigm in the government’s population policy compared to the previous rounds. The Fourth Basic Plan presents a vision of creating “a sustainable society where all generations are happy together,” acknowledging that current birth rates and demographic phenomena are the outcomes of individual choices and adaptation, not of governmental regulation and control. This plan makes it clear that the focus will be on bringing about structural changes at the individual, family, regional, and social levels. This is indeed a shift in policy direction and will be a welcome shift new beginning, no matter how overdue the change is.

The new plan envisions a society where we “work and care together” as a strategy to stop low birth [2]. Any woman who would like to work should be able to find employment, and not forced to discontinue their careers, endure job instability, or work in a low-paying job. Key strategies include creating gender-equal labor conditions so that all genders can plan stable careers, as well as improving current maternity/paternity leave policies so that everyone can share the labor of work and parenting. The COVID-19 crisis has shed light on the fact that the labor of caregiving plays a key role in sustaining families and society. Housekeeping, childcare, and elderly caregiving should not be underestimated or taken for granted as women’s tasks. Homes, businesses, and local communities should all contribute to transform Korean society into one where people truly “work and care together.”

It is also notable that the new plan penciled in the tasks of “guaranteeing the rights to sex and reproduction over the lifetime” and “legally embracing various types of families” in its strategies [2]. The government should support healthcare services from the point a woman gets pregnant, as well as providing health coverage for the entire life cycle, from adolescence to old age, covering sexual health, menstrual health, contraceptives, abortion, gender-based violence, sexually transmitted infections, and cancer control and prevention. Furthermore, Korean law only protects families that consist of “relationships based on marriage, blood, and adoption,” while France and Germany have expanded their legal boundaries of family to embrace unions that emotionally and financially support each other without official marriage, through the Civil Solidarity Pact and the Life Companion Law, respectively [10]. In 2019, 2.3% births in Korea were out of wedlock [1], the highest percentage in the country’s history but the lowest among Organisation for Economic Co-operation and Development (OECD) nations—along with the lowest TFR. Korea should join the ranks of the global community in banning discrimination against diverse types of families and should create an environment where all types of families and their children are respected and embraced.

The issue of low birth and aging population is caused by a combination of complex social issues that exist in Korean society. It is true that this phenomenon may affect our country's socio-economic structure. As we see many countries overcoming the issue of low birth, Korea should restore trust within its society, so that everyone is respected and able to choose their own lifestyle, rather than focusing on this crisis that fuels public anxiety. Hopefully, Korea will evolve into a society where all residents can protect their own family and exercise reproductive rights with enhanced gender equality and quality of life. A stronger focus on women’s health is needed within public health and health-related policy. Moreover, as the impact of sexual and reproductive health on women’s lives and quality of life is paramount, active education, research, and policy development is more urgent than ever.
